# Numerical Analysis and Strain Monitoring of the Curing Process in Ring-Shaped CFRP Components

**DOI:** 10.3390/polym18121447

**Published:** 2026-06-10

**Authors:** Yanhui Tian, Benjie Ding, Jianke Du, Minghua Zhang

**Affiliations:** School of Mechanical Engineering and Mechanics, Ningbo University, Ningbo 315211, China; tyh19032170525@163.com (Y.T.); dingbenjie@nbu.edu.cn (B.D.); dujianke@nbu.edu.cn (J.D.)

**Keywords:** CFRP, cure kinetics, multi-field coupling, curing deformation, numerical simulation, optical fiber monitoring

## Abstract

Multi-field coupled numerical analysis and strain monitoring experiments were conducted for the curing process of a ring-shaped CFRP component. The curing kinetics and mechanical properties of LD-2184 epoxy resin were characterized using non-isothermal DSC, tensile testing, and CTE measurements. The curing reaction follows a single-stage autocatalytic mechanism with an activation energy of 54.73 kJ·mol^−1^. A piecewise curing kinetics equation was established. The elastic modulus of the fully cured resin is 2.810 GPa, and the coefficient of thermal expansion is 6.060 × 10^−5^ K^−1^. Composite ring specimens were fabricated using a wet winding process. FBG sensors were embedded to monitor axial strain during curing. A coupled numerical model was developed that includes heat conduction, curing kinetics, and curing deformation. ABAQUS was used to simulate the curing process of the composite ring. The results show a temperature gradient within the filament-wound layer. Thermo-chemical strain is similar between inner and outer regions. Total strain varies along the thickness due to mold constraint. Residual stress is governed by resin chemical shrinkage and thermal contraction during cooling. The difference between measured and simulated strain is 7.15%, which supports the validity of the multi-field coupled curing model.

## 1. Introduction

Resin-based carbon fiber composite (CFRP) is a composite material system that uses thermosetting or thermoplastic resin as the matrix and carbon fiber and its fabrics as reinforcement. It exhibits high specific strength, high specific stiffness, and strong adaptability to extreme environments such as high temperature and high corrosion [1,2]. In the aerospace field, which imposes stringent requirements on load-bearing structures [3], components fabricated from CFRP can reduce weight and loading while maintaining structural strength and safety. The mechanical properties remain stable under extreme conditions. Composite shells made from this material have become key components of solid rocket motors.

Despite these advantages, several challenges persist. During curing, thermal effects, resin chemical shrinkage, and the mismatch in coefficient of thermal expansion (CTE) between the composite and the mold [4] induce residual stress and curing deformation. Residual stress reduces the load-bearing capacity of components. Curing deformation leads to dimensional deviations from design specifications and affects molding quality. Empirical trial-and-error approaches have traditionally been used to adjust curing processes and reduce deformation [5]. This approach remains inefficient and costly, which limits its application in modern production. Advances in computer technology have enabled the widespread use of finite element simulation in composite design and manufacturing, providing an effective tool for analyzing curing behavior in resin-based composites.

During the curing of thermosetting resins, irreversible chemical crosslinking reactions occur. These reactions release heat and alter the material state and mechanical properties, which affect the overall performance of the composite. Investigation of curing behavior is essential for numerical analysis of composite curing. Differential scanning calorimetry (DSC), a widely used thermal analysis method, has been extensively applied to study resin curing kinetics. Lee et al. [6] determined the curing kinetic parameters of 3501-6 resin using DSC. Salla et al. [7] investigated the curing kinetics of unsaturated polyester resin using DSC and compared the effects of dynamic and isothermal testing protocols. Chu et al. [8] examined the curing reaction characteristics of a DMBA-catalyzed acrylic/epoxy resin system through combined dynamic and isothermal DSC experiments. Kim et al. [9] analyzed the isothermal curing kinetic model, heat of reaction, and glass transition temperature of AS4/3501-6 prepreg using DSC. Several studies have also examined the evolution of temperature and degree of cure in relation to thermophysical properties such as thermal conductivity, density, and specific heat capacity during curing [10,11]. Pedersen et al. [12] used MDSC to investigate the variation in thermal diffusivity of epoxy resin during curing. Struzziero et al. [13] developed an experimental apparatus for measuring thermal conductivity in a cylindrical container and analyzed the evolution of thermal conductivity in three aerospace-grade epoxy resins during curing.

In numerical simulations of composite curing, many studies have focused on the evolution of curing degree, temperature field, and thermo-chemical coupling effects [14,15]. Behzad et al. [16] developed a nonlinear transient heat transfer analysis method by incorporating a curing kinetic model into a finite element framework and performed three-dimensional simulations of an automotive mirror housing to predict temperature and degree of cure during molding. Abdelal et al. [17] established a thermo-chemical coupling model and calculated temperature and degree of cure distributions in a unidirectional composite during autoclave curing using implicit and explicit finite element methods. Brauner et al. [18] conducted thermo-chemical modeling to analyze the curing behavior of a composite aircraft fuselage frame during manufacturing.

The development of constitutive models for composite curing has progressed through continuous refinement. Bogetti et al. [19] proposed a curing-hardening instantaneous linear elastic model that describes the variation in resin modulus with degree of cure, referred to as the CHILE(α) model. Johnston et al. [20] reported that the resin modulus shifts with temperature during curing and proposed the CHILE($T_{g}$) model, in which the modulus depends on the glass transition temperature. Subsequent studies incorporated viscoelastic behavior and stress relaxation during curing, introducing viscoelastic constitutive relations into simulations of curing deformation in composites [21,22,23,24,25]. These models provide a more accurate description of resin property evolution, yet they require extensive experimental data for parameter identification and involve high computational cost. The CHILE(α) model remains extensively used in engineering applications due to its simpler parameter determination.

Numerical simulations of curing in resin-based composites have primarily focused on laminates and L-shaped structures [26,27,28]. Studies on axisymmetric structures such as rings and shells remain limited. Fiber Bragg Grating (FBG) sensors have been widely used in composite structural health monitoring due to their small size, immunity to electromagnetic interference, and ease of embedding [29,30]. Based on this background, the present study investigates a ring-shaped CFRP component through numerical simulation and curing monitoring of the thermo-chemo-mechanically coupled curing process. The curing kinetics and mechanical properties of LD-2184 epoxy resin were first characterized experimentally. Composite ring specimens embedded with FBG sensors were fabricated using a wet winding process to monitor strain evolution in real time during curing. Multi-field coupled numerical analysis of the curing process was then performed using ABAQUS (v2017). Simulation results were compared with FBG monitoring data to assess the validity of the numerical approach and to identify sources of error. This process–simulation framework provides a reference for evaluating the curing quality of ring-shaped composite components and can serve as a predictive tool for curing process optimization and mold design of such components in industrial applications. Furthermore, it lays a foundation for future investigations into the curing process of more complex shell and vessel structures.

## 2. Characterization of Resin Properties

### 2.1. Preparation of Resin Matrix

Materials: LD-2184 epoxy resin (DGEBA) and curing agent (amine) were purchased from Shijiazhuang Liding Electronic Materials Co., Ltd. (Shijiazhuang, China). The LD-2184 resin and curing agent were mixed at a mass ratio of 100:53 until a uniform mixture was obtained. The mixture was then placed in a DZF-6090AB vacuum oven for degassing at room temperature.

### 2.2. Methods

#### 2.2.1. Non-Isothermal DSC Test

Non-isothermal DSC tests were conducted to examine the curing reaction characteristics of LD-2184 resin using a Netzsch DSC-200 F3 differential scanning calorimeter. A small amount of resin was weighed and placed in an aluminum crucible. The sample mass did not exceed 25 mg to prevent overflow during thermal expansion. The temperature range was set from 25 to 300 °C. Heating was performed in a nitrogen atmosphere at rates of 5, 10, 15, and 20 °C·min^−1^, and the corresponding DSC curves were recorded.

#### 2.2.2. Tensile Test

**Figure 1:**
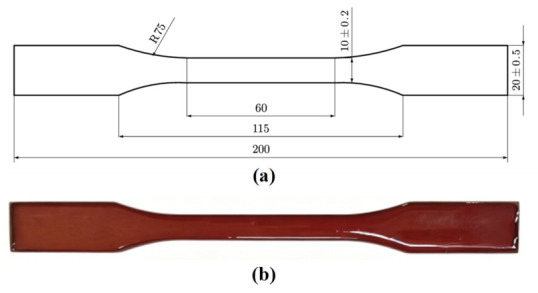
(**a**) Dimension diagram of tensile specimen (unit: mm); (**b**) tensile specimen.

Tensile tests were conducted on cured resin specimens to determine the elastic modulus required for numerical analysis. The specimen geometry is shown in Figure 1, and the dimensions comply with the relevant standard [31]. A total of four specimens were tested. Testing was performed using an AGS-X Shimadzu benchtop universal testing machine at a loading rate of 2 mm·s^−1^.

#### 2.2.3. CTE Test

Thermomechanical analysis (TMA) was performed on cured resin samples to evaluate dimensional stability and determine the coefficient of thermal expansion (CTE). A total of three samples with dimensions of (12–25) × 5 × 5 mm^3^ were tested, and a probe force of 0.05 N was applied. The tests were conducted in accordance with the relevant standard [32]. The samples were held at 20 °C for 3 min and then heated at a rate of 5 °C·min^−1^. Thermal expansion curves were recorded for subsequent fitting and analysis.

### 2.3. Test Results and Discussion

**Figure 2:**
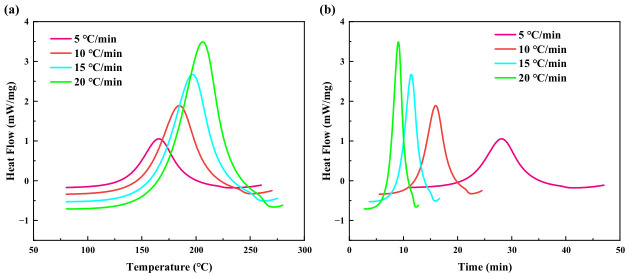
Heat flow of LD-2184 resin at different heating rates: (**a**) variation with temperature; (**b**) variation with time.

The variation in heat flow of LD-2184 resin with temperature and time at different heating rates is shown in Figure 2a,b. Each dataset represents the average of three replicate tests conducted at the same heating rate. The DSC curves exhibit a single exothermic peak at all heating rates, which indicates a single-stage curing reaction. As the heating rate increases, both the peak heat flow and peak temperature increase, while the reaction time decreases. The exothermic peak shifts toward higher temperatures, which reflects an exothermic lag in the curing reaction.

Characteristic parameters derived from the DSC results, including peak temperature, peak heat flow, and total heat release of cure obtained from the area under the exothermic peak, are summarized in Table 1. The total heat release values were averaged to obtain a final reaction heat of 593.22 J·g^−1^ for LD-2184 resin.

The degree of cure of the epoxy resin is defined as:

(1) $$\alpha =\frac{H_{T}}{H_{U}},$$

where $H_{T}$ represents the heat released at a given time, obtained by integrating the heat flow curve in Figure 2b over time, and $H_{U}$ represents the total heat release listed in Table 1.

**Figure 3:**
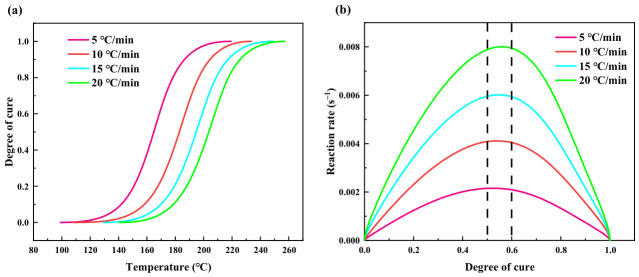
(**a**) Variation in curing degree with temperature; (**b**) variation in curing rate with curing degree.

Figure 3a shows the variation in the degree of cure with temperature for LD-2184 resin. The curves exhibit an S-shaped trend. In the intermediate stage, rapid curing increases the crosslink density of molecular chains, which leads to a sharp increase in the degree of cure. The curing reaction rate is obtained by differentiating the degree of cure with respect to time. Figure 3b shows the relationship between curing rate and degree of cure. The curing rate increases with increasing heating rate. All curves exhibit a parabolic profile, with peak values in the degree of cure range of 0.5–0.6. This behavior indicates that the curing kinetics is independent of heating rate and follows an autocatalytic mechanism. An autocatalytic kinetics model is adopted to describe the curing reaction of LD-2184 resin.

The activation energy of the curing reaction was determined through mathematical fitting based on the Kissinger equation and the Ozawa equation [33].

The Kissinger equation is expressed as follows:

(2) $$ln\left(\frac{\tau }{T_{P}^{2}}\right)=ln\left(\frac{AR}{E_{a}}\right)-\frac{E_{a}}{R}\frac{1}{T_{P}},$$

where *R* is the ideal gas constant, taken as 8.314 J·(mol·K)^−1^, *A* is the frequency factor, with units of S^−1^, $E_{a}$ is the activation energy of the curing reaction being determined, and τ and $T_{p}$ represent the heating rate and the corresponding peak temperature, respectively, with values shown in Table 1.

The Ozawa equation is expressed as follows:

(3) $$\frac{d(ln\;\tau )}{d(\frac{1}{T_{P}})}=-1.052\frac{E_{a}}{R},$$

The parameters have the same definitions as in Equation (2).

**Figure 4:**
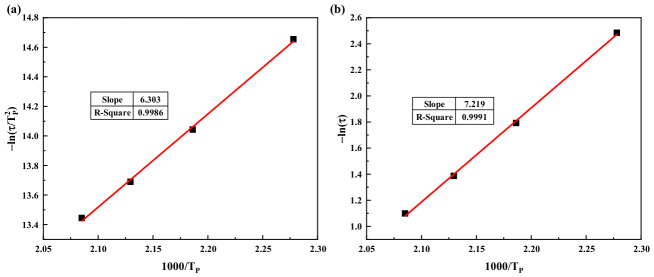
Fitting of activation energy: (**a**) Kissinger equation; (**b**) Ozawa equation.

Based on Table 1, a plot of $-ln(\tau /T_{P}^{2})$ versus 1000/$T_{P}$ was constructed and fitted linearly, as shown in Figure 4a. The slope of the fitted line is 6.303, and the coefficient of determination is 0.9986, which indicates good agreement. The activation energy calculated from Equation (2) is 52.40 kJ·mol^−1^. A plot of −ln(τ) versus 1000/$T_{P}$ was then constructed and fitted, as shown in Figure 4b. The slope is 7.219 with a value of 0.9991. The activation energy obtained from Equation (3) is 57.05 kJ·mol^−1^. The average of the two results gives a final activation energy of 54.73 kJ·mol^−1^ for LD-2184 resin.

A curing kinetics model for epoxy systems with autocatalytic behavior is expressed as:

(4) $$\frac{d\alpha }{dt}=k\left(T\right)\alpha^{m}{\left(1-\alpha \right)}^{n},$$

where *m* and *n* are the reaction orders; $k\left(T\right)$ follows the Arrhenius relation:

(5) $$k\left(T\right)=A\cdot exp\left(-\frac{E_{a}}{RT}\right),$$

**Figure 5:**
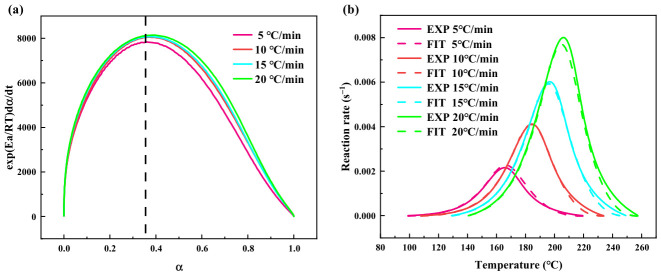
(**a**) Relationship between $exp\left(E_{a}/RT\right)d\alpha /dt$ and α; (**b**) comparison of calculated and experimental curing rate curves.

The relationship between $exp\left(E_{a}/RT\right)d\alpha /dt$ and the degree of cure α is shown in Figure 5a. The extrema of all curves occur near α=0.35. A piecewise nonlinear fit was applied using α=0.35 as the boundary. The parameters *A*, *m*, and *n* were obtained for each heating rate and then averaged. The resulting curing kinetics model is:

(6) $$\frac{d\alpha }{dt}=\left\{\begin{array}{c} 15243\times exp(-\frac{54730}{8.314T})\alpha^{0.424}{\left(1-\alpha \right)}^{0.415},\alpha \leq 0.35\\ 33763\times exp(-\frac{54730}{8.314T})\alpha^{0.829}{\left(1-\alpha \right)}^{1.321},\alpha \geq 0.35 \end{array}\right.,$$

Equation (6) was used to calculate the average curing rate curves for each heating rate. Figure 5b compares the calculated and experimental curves. The results show good agreement, and the overall trend of the curing rate is consistent.

**Figure 6:**
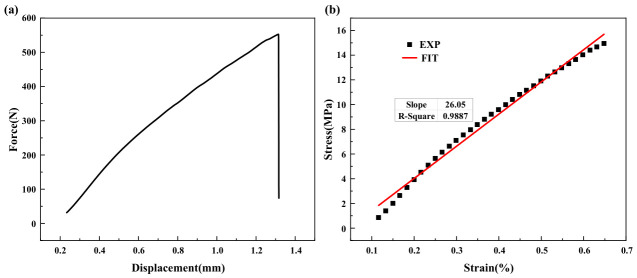
(**a**) Force–displacement curve (one specimen); (**b**) stress–strain curve and fitting result.

Figure 6a shows a representative force–displacement curve of one specimen obtained from the tensile test. The stress was calculated by dividing the force by the cross-sectional area of the specimen, and the nominal strain was obtained by dividing the displacement by the length of the tensile section. The nominal strain was then converted to true strain according to Equation (7). Figure 6b shows the corresponding stress–strain curve derived from the linear region, along with the linear fit. The slope of 26.05 indicates that the elastic modulus of the fully cured resin is 2.605 GPa. The average elastic modulus obtained from the four specimens is 2.810 ± 0.140 GPa (mean ± SD).

(7) $$\varepsilon_{T}=ln(1+\varepsilon_{N}),$$

where $\varepsilon_{T}$ is true strain, and $\varepsilon_{N}$ is nominal strain.

**Figure 7:**
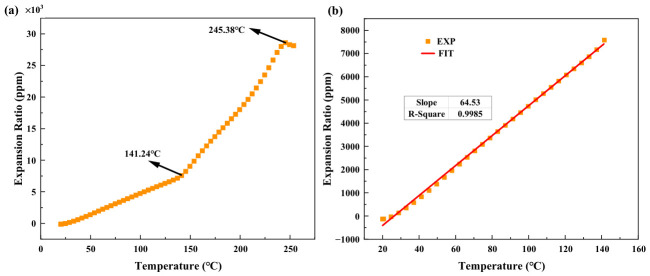
(**a**) Thermal expansion curve (one sample); (**b**) CTE fitting.

Figure 7a shows a representative thermal expansion curve of one sample obtained from CTE testing. The resin exhibits distinct thermal stability across different temperature ranges. In the low-temperature range, the expansion increases approximately linearly. When the temperature exceeds 141.24 °C, the slope of the curve increases and the expansion rate accelerates. This change corresponds to the transition from a glassy state to a rubbery state, which reduces resistance to thermal deformation. When the temperature exceeds 245.38 °C, the curve decreases, which indicates the onset of melting. The test was terminated at this stage to prevent contamination of the instrument by molten material. The curing temperatures in this study were below 160 °C. The thermal expansion data in the range of 20 °C to 141.24 °C were selected for linear fitting. The fitting result is shown in Figure 7b. The slope of the fitted curve is 64.53, which corresponds to a CTE of 6.453 × 10^−5^ K^−1^. The average CTE obtained from the three samples is (6.060 ± 0.419) × 10^−5^ K^−1^. The average result was used to determine the CTE as an input parameter for numerical simulation.

## 3. Experiment and Numerical Simulation

### 3.1. Curing Monitoring Experiment

**Figure 8:**
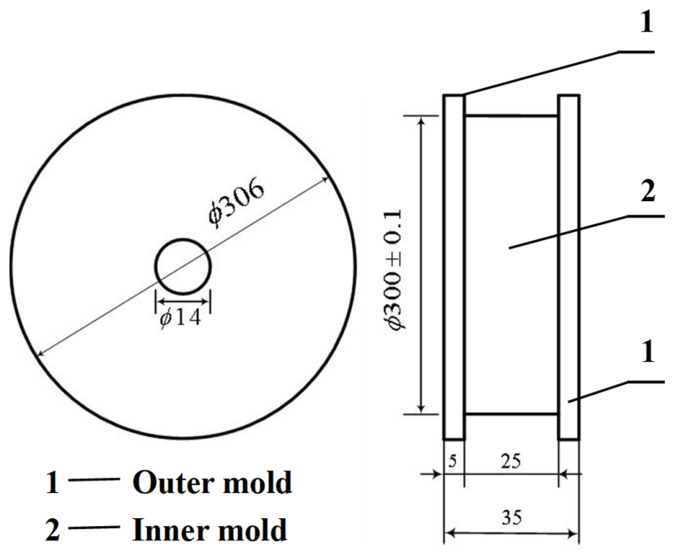
Geometric dimensions of the winding mold (unit: mm).

Figure 8 shows the geometric dimensions of the winding mold for the composite ring. The mold consists of an inner mold and an outer mold, and the ring is formed by winding carbon fiber on the inner mold. Composite ring specimens were fabricated using a wet winding process on a horizontal CNC winding machine. The raw material was T800/LD-2184 prepreg, and hoop winding was adopted. The fiber tow width was 8.1 mm, and the winding tension was maintained at 45 N. A total of 12 layers were wound, resulting in a thickness of 2.4 mm. The fiber volume fraction of the fabricated ring specimens is 50%.

**Figure 9:**
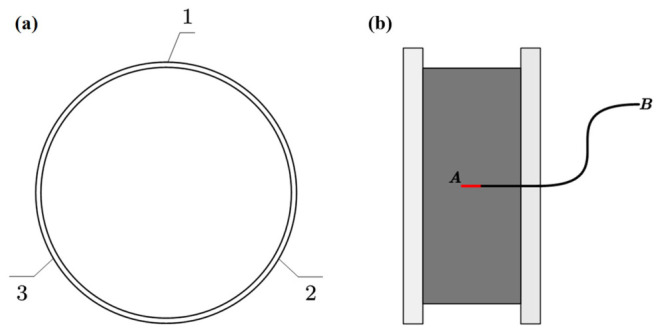
(**a**) FBG embedding position; (**b**) FBG wiring direction.

To monitor axial strain during curing, Fiber Bragg Grating (FBG) sensors (Fibercore) were embedded between the 10th and 11th layers. As shown in Figure 9, three sensors were arranged uniformly along the circumference. The embedding direction was perpendicular to the fiber direction. The grating region at point A has a length of 5 mm. Point B is connected to the FBG interrogator to record the central wavelength variation.

The central wavelength shift of an FBG depends on both strain and temperature. A single FBG cannot separate these effects. A temperature sensor was mounted on the surface of each specimen. The sensing region was enclosed in a capillary tube to isolate it from strain. During monitoring, the uncoated FBG sensor records wavelength shifts caused by both strain and temperature. The temperature-induced component measured by the temperature sensor is removed to obtain strain:

(8) $$\varepsilon =\frac{\lambda -\lambda_{0}-(\lambda^{T}-\lambda_{0}^{T})\times \frac{1}{a}\times b}{K},$$

where λ is the measurement wavelength of the FBG grating, $\lambda_{0}$ is the initial wavelength of the FBG grating, $\lambda^{T}$ is the measurement wavelength of the temperature sensor grating, $\lambda_{0}^{T}$ is the initial wavelength of the temperature sensor grating, *a* is the temperature sensitivity coefficient of the temperature sensor, *b* is the temperature sensitivity coefficient of the FBG, and *K* is the strain sensitivity coefficient of the FBG. Based on the parameter data provided by the supplier, when the temperature is below 100 °C, *a* is set to 9.96 pm·°C^−1^; when the temperature is above 100 °C, *a* is set to 12.70 pm·°C^−1^; and the values for *b* and *K* are 10 pm·°C^−1^ and 1.2 pm·°C^−1^, respectively.

**Figure 10:**
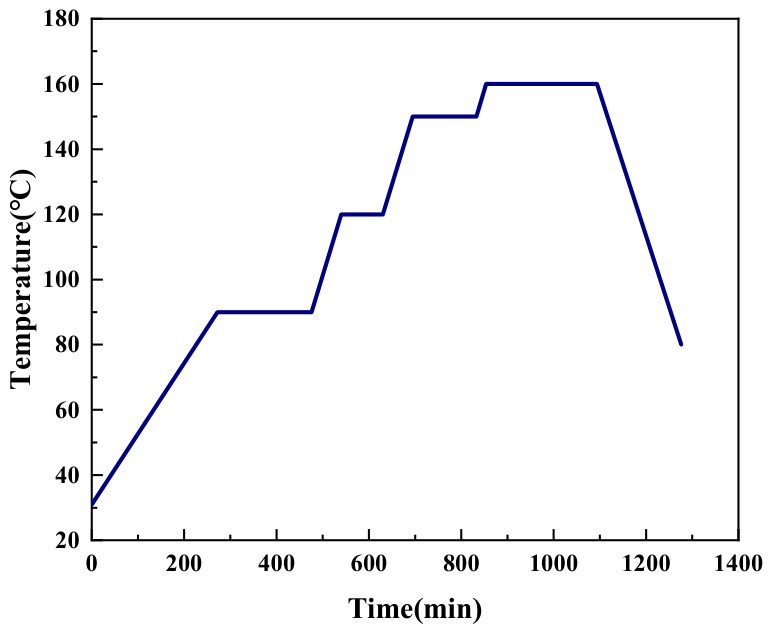
Curing temperature profile.

Three ring specimens were prepared and placed horizontally in a curing oven. The FBG and temperature sensor leads were connected to an interrogator outside the oven. The temperature program followed the curing profile shown in Figure 10, and strain monitoring was conducted during the curing process.

### 3.2. Curing Coupled Model

#### 3.2.1. Thermo-Chemical Coupled Model

The temperature field during the curing of resin-based composites is governed by heat conduction and the exothermic curing reaction. The temperature field also affects the evolution of the degree of cure. The curing process is treated as a thermo-chemical coupled process. The temperature field in anisotropic composites is described by the three-dimensional Fourier heat conduction equation [27]:

(9) $$\rho C\frac{\partial T}{\partial t}=k_{11}\frac{\partial^{2}T}{\partial x^{2}}+k_{22}\frac{\partial^{2}T}{\partial y^{2}}+k_{33}\frac{\partial^{2}T}{\partial z^{2}}+Q,$$

where ρ and C are the density and specific heat capacity of the composite, respectively, $k_{ii}$ is the thermal conductivity along the principal directions, *t* is time, and *Q* is the internal heat source:

(10) $$Q=\rho_{r}V_{r}H_{U}\frac{d\alpha }{dt},$$

where $\rho_{r}$ is the resin density, $V_{r}$ is the resin volume fraction, $H_{U}$ is the reaction heat of the resin as determined by DSC testing, and dα/dt is the curing rate calculated from Equation (6).

#### 3.2.2. Curing Deformation Model

Curing deformation in composites arises from thermal effects and chemical shrinkage. The constitutive relation that accounts for thermo-chemical strain is expressed as [34]:

(11) $$\left\{\sigma \right\}=\left[C\right]\left(\left\{\varepsilon_{tot}\right\}-\left\{\varepsilon_{tc}\right\}\right),$$

where σ is stress, $\left[C\right]$ is the stiffness matrix, and $\varepsilon_{tot}$ and $\varepsilon_{tc}$ are total strain and thermo-chemical strain, respectively.

The elastic modulus of the resin varies with the degree of cure. This variation is described by the CHILE(α) model [10]:

(12) $$E_{r}=\left\{\begin{array}{ll} E_{r}^{0} & \alpha \leq \alpha_{gel}\\ \left(1-\alpha_{mod}\right)E_{r}^{0}+\alpha_{mod}E_{r}^{\infty } & \alpha_{gel}\leq \alpha \leq \alpha_{diff}\\ E_{r}^{\infty } & \alpha \geq \alpha_{diff} \end{array}\right.,$$

where $\alpha_{mod}=\left(\alpha -\alpha_{gel}\right)/\left(\alpha_{diff}-\alpha_{gel}\right)$; $\alpha_{gel}$ and $\alpha_{diff}$ are the degree of cure at the resin’s gel point and the degree of cure upon completion of curing, respectively. $E_{r}^{0}$ and $E_{r}^{\infty }$ are the elastic modulus of the resin before and after curing, respectively, with $E_{r}^{\infty }$ typically taken as 1000 times $E_{r}^{0}$. Based on the tensile test results, $E_{r}^{\infty }=2.810$ GPa, and we assume $\alpha_{gel}=0$ and $\alpha_{diff}=1$.

The composite is treated as a homogeneous material composed of carbon fibers and a resin matrix. Equivalent elastic constants are calculated using mesomechanics relations [35]:

(13) $$\left\{\begin{array}{c} E_{1}=E_{1}^{f}V_{f}+E_{r}V_{r}+\frac{4(\nu_{r}-\nu_{12}^{f}{)}^{2}K_{r}K_{f}G_{r}V_{f}V_{r}}{(K_{f}+G_{r})K_{r}+(K_{f}-K_{r})G_{r}V_{f}}\\ E_{2}=E_{3}=\frac{1}{1/(4K_{T})+1/(4G_{23})+{\left(\nu_{12}\right)}^{2}/E_{1}}\\ G_{12}=G_{13}=G_{r}\left[\frac{(G_{12}^{f}+G_{r})+(G_{12}^{f}-G_{r})V_{f}}{(G_{12}^{f}+G_{r})-(G_{12}^{f}-G_{r})V_{f}}\right]\\ G_{23}=\frac{G_{r}\left[(G_{23}^{f}+G_{r})K_{r}+2G_{23}^{f}G_{r}+(G_{23}^{f}-G_{r})K_{r}V_{f}\right]}{(G_{23}^{f}+G_{r})K_{r}+2G_{23}^{f}G_{r}-(K_{r}+2G_{r})(K_{r}+2G_{r})V_{f}} \end{array}\right.,$$

The longitudinal and transverse Poisson’s ratios are expressed as:

(14) $$\left\{\begin{array}{c} \nu_{12}=\nu_{13}=\nu_{12}^{f}V_{f}+\nu_{r}V_{r}+\frac{(\nu_{r}-\nu_{12}^{f})(K_{r}-K_{f})G_{r}V_{f}V_{r}}{(K_{f}+G_{r})K_{r}+(K_{f}-K_{r})G_{r}V_{f}}\\ \nu_{23}=1-\frac{E_{1}E_{2}+4{{\left(\nu_{12}\right)}^{2}E}_{1}K_{T}}{2E_{1}K_{T}} \end{array}\right.,$$

$K_{f}$ and $K_{r}$ are the isotropic bulk moduli of carbon fiber and resin, respectively, and $K_{T}$ is the effective bulk modulus of the lamina, which can be expressed as follows:

(15) $$\left\{\begin{array}{c} K_{f}=\frac{E_{1}^{f}E_{2}^{f}}{2(1-\nu_{23}^{f})E_{1}^{f}-4(\nu_{12}^{f}{)}^{2}E_{2}^{f}}\\ K_{r}=\frac{E_{r}}{2(1-\nu_{r})-4{\left(\nu_{r}\right)}^{2}}\\ K_{T}=\frac{(K_{f}+G_{r})K_{r}-(K_{f}-K_{r})G_{r}V_{f}}{(K_{f}+G_{r})-(K_{f}-K_{r})V_{f}} \end{array}\right.,$$

where subscripts 1, 2, and 3 represent the three principal axis directions; *f* and *r* represent carbon fiber and resin, respectively; and *V* represents the volume fraction.

Thermo-chemical strain is the sum of thermal strain and chemical strain:

(16) $$\left\{\varepsilon_{tc}\right\}=\left\{\varepsilon_{th}\right\}+\left\{\varepsilon_{ch}\right\},$$

where $\varepsilon_{th}$ and $\varepsilon_{ch}$ are thermal strain and chemical strain, respectively.

Thermal strain can be calculated as follows:

(17) $$\varepsilon_{th}=\phi_{i}\Delta T,$$

where ΔT is the temperature increment; $\phi_{i}$ is the CTE of the composite along the three principal axes [35]:

(18) $$\phi_{1}=\frac{V_{f}\phi_{1}^{f}E_{1}^{f}+V_{r}\phi_{r}E_{r}}{V_{f}E_{1}^{f}+V_{r}E_{r}},$$

(19) $$\phi_{2}=\phi_{3}=V_{f}(\phi_{2}^{f}+\nu_{12}^{f}\phi_{1}^{f})+V_{r}(1+\nu_{r})\phi_{r}-(\nu_{12}^{f}V_{f}+\nu_{r}V_{r})\frac{V_{f}\phi_{1}^{f}E_{1}^{f}+V_{r}\phi_{r}E_{r}}{V_{f}E_{1}^{f}+V_{r}E_{r}},$$

Carbon fibers do not undergo chemical reactions during curing. Chemical strain arises from resin shrinkage. The curing shrinkage strain of the resin is expressed as [35]:

(20) $$\varepsilon_{sh}^{r}=\sqrt[{3}]{1+\Delta v}-1,$$

where Δv is the chemical shrinkage related to the degree of cure:

(21) $$\Delta v=\Delta \alpha \cdot v_{sh},$$

where $v_{sh}$ is the total volume shrinkage of the resin after curing.

The chemical strain of the composite is then given by [35]:

(22) $$\varepsilon_{1ch}=\frac{\varepsilon_{sh}^{r}E_{r}V_{r}}{E_{1}^{f}V_{f}+E_{r}V_{r}},$$

(23) $$\varepsilon_{2ch}=\varepsilon_{3ch}=V_{r}(1+\nu_{r})\varepsilon_{sh}^{r}-(\nu_{12}^{f}V_{f}+\nu_{r}V_{r})\frac{\varepsilon_{sh}^{r}E_{r}V_{r}}{E_{1}^{f}V_{f}+E_{r}V_{r}},$$

### 3.3. Finite Element Modeling

**Figure 11:**
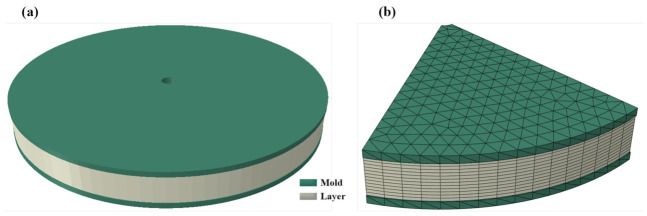
(**a**) Finite element model; (**b**) 1/6 sector model with mesh.

A finite element model was established in ABAQUS, as shown in Figure 11a. The winding layers are composed of T800 carbon fiber and LD-2184 epoxy resin, and their mechanical properties are listed in Table 2. Due to limitations of the experimental conditions, k, ρ, and C of the resin and carbon fiber are taken from references [36]. Each layer has a thickness of 0.2 mm, with a total of 12 layers. The winding pattern is circumferential with a winding angle of 90°. The mold material is 30CrMnSiA steel, and its mechanical properties are given in Table 3. The symmetry of the ring was considered to reduce computational cost. A 1/6 sector model was adopted and discretized, as shown in Figure 11b. The mold was meshed using C3D10MT elements, which are second-order thermomechanically coupled tetrahedral elements. The winding layers were meshed using C3D8T elements, which are thermomechanically coupled hexahedral elements.

**Figure 12:**
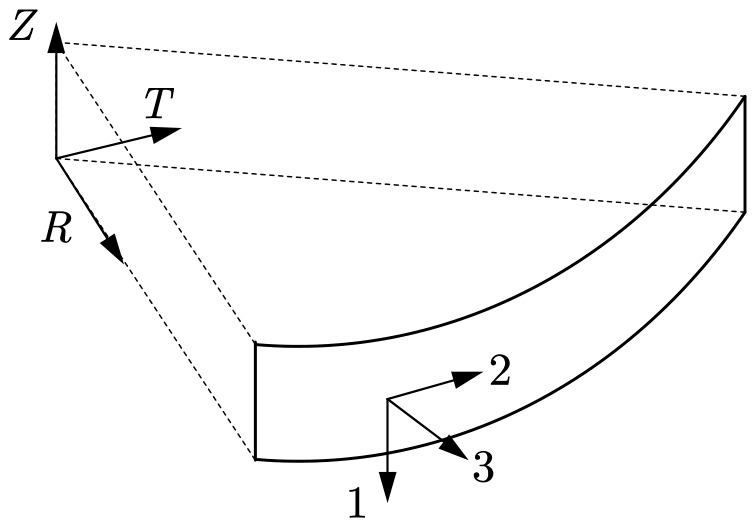
Local coordinate system of the composite ring.

In the ABAQUS model, material orientation must be defined for the composite layers. A local coordinate system was assigned based on the cylindrical coordinate system (R, T, Z), as shown in Figure 12. The system was rotated by 90° about the 2-axis. After rotation, axis 1 corresponds to the axial direction, axis 2 to the fiber direction, and axis 3 to the thickness direction. In this coordinate system, the angle between the material orientation and the principal axis at each element corresponds to the 90° winding angle.

Boundary conditions and loading were defined as follows. The interface between the winding layers and the 30CrMnSiA mold was defined as a tied constraint. Displacement in the Y-direction was fixed at the bottom surface. Symmetry boundary conditions were applied on both lateral faces of the 1/6 model in the circumferential direction to eliminate rigid-body motion. The curing temperature field, shown in Figure 10, was applied to the outer surface.

**Figure 13:**
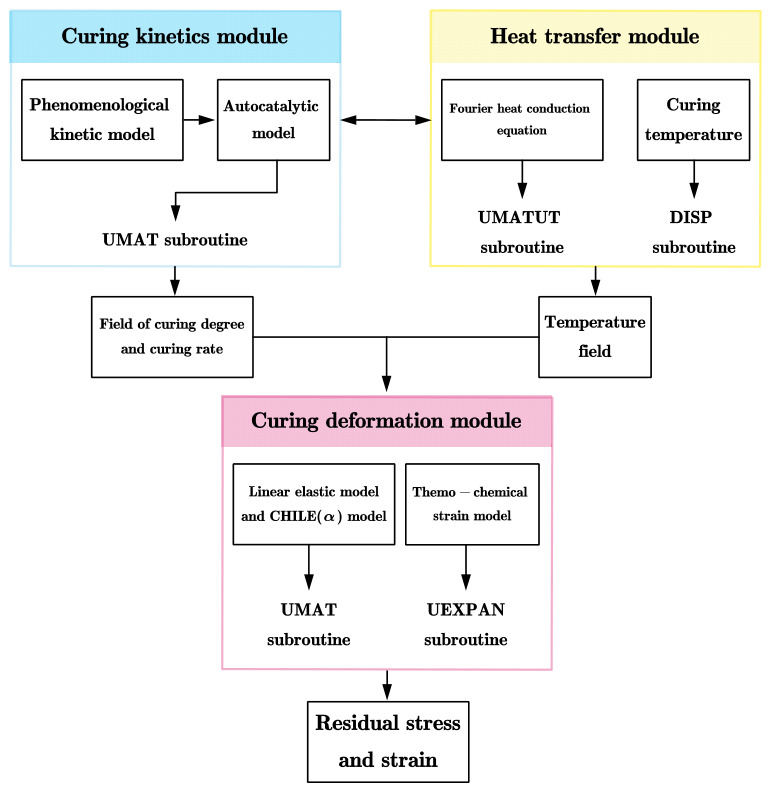
Flowchart of coupled curing calculation.

Multi-field coupled simulation of the curing process was implemented through ABAQUS user subroutines. UMAT defines the constitutive model and curing kinetics, computes equivalent elastic constants, forms the stiffness matrix, and performs coordinate transformation based on fiber orientation at integration points. UMATHT governs heat transfer. DISP applies the curing temperature field as a prescribed load. UEXPAN calculates thermo-chemical strain. The computational procedure is illustrated in Figure 13, which includes the heat transfer module, curing kinetics module, and curing deformation module.

## 4. Results and Discussion

### 4.1. Simulation Result Analysis

#### 4.1.1. Composite Mechanical Properties

As the curing reaction proceeds, the resin modulus increases, leading to a continuous rise in the homogenized modulus calculated by Equation (13). At the same time, the CTE of the composite also changes continuously. Once the curing process is essentially complete (α=1), the mechanical properties of the composite tend to stabilize, as shown in Table 4.

#### 4.1.2. Temperature and Curing Degree Analysis

**Figure 14:**
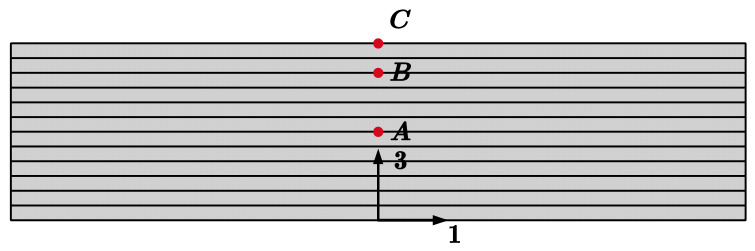
Position of observation points within the cross-section of the filament-wound layer.

Three observation points (A, B, and C) were selected on the cross-section of the filament-wound layer, as shown in Figure 14. These points are located along the thickness direction (axis 3) at the mid-section. Point A is at the center, point B is positioned between the 10th and 11th layers, and point C is located on the outer surface.

**Figure 15:**
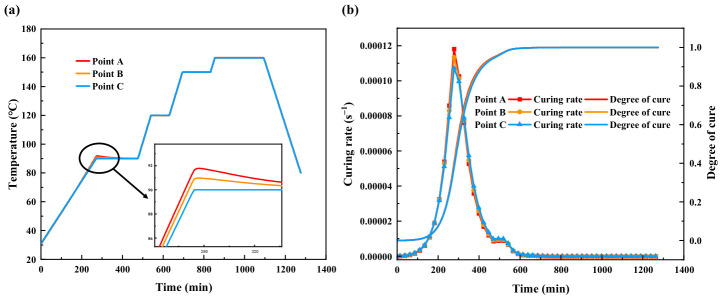
Points A, B, and C: (**a**) temperature evolution; (**b**) degree of cure and curing rate.

**Figure 16:**
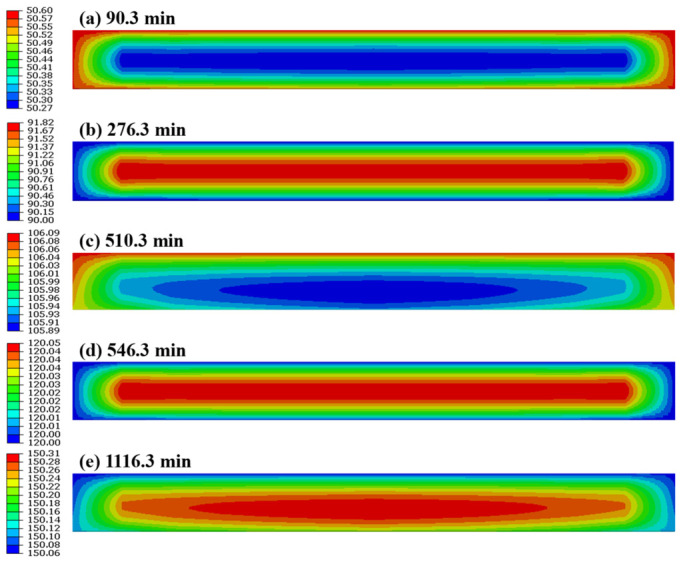
Temperature distribution within the cross-section of the filament-wound layer: (**a**) 90.3 min; (**b**) 276.3 min; (**c**) 510.3 min; (**d**) 546.3 min; (**e**) 1116.3 min.

Figure 15a shows the temperature evolution at points A, B, and C during curing. Figure 15b shows the corresponding degree of cure and curing rate. Figure 16 presents the temperature distribution across the cross-section at different times. During the initial heating stage, heat is transferred from the exterior to the interior, which results in a lower internal temperature compared to the outer region (Figure 16a). As the temperature increases and reaches the first holding stage, the resin undergoes an intense curing reaction. The released heat raises the internal temperature above the external temperature, with a first peak of 91.82 °C at 276.3 min (Figure 16b). The curing rate at point A exceeds those at points B and C due to heat accumulation, as shown in Figure 15b. During the second heating stage, delayed heat transfer leads to a lower internal temperature relative to the exterior (Figure 16c). In the second holding stage, heat accumulation again increases the internal temperature, and the high-temperature region shifts inward. A second peak of 120.05 °C occurs at 546.3 min (Figure 16d). After approximately 630 min, the curing reaction approaches completion, and the exothermic heat decreases. No distinct high-temperature region is observed in the interior during the subsequent stages. During cooling, heat is transferred from the interior to the exterior, which results in a higher internal temperature (Figure 16e). The temperature difference across the thickness remains small throughout the process due to the relatively thin structure of the ring.

#### 4.1.3. Residual Strain and Residual Stress Analysis

**Figure 17:**
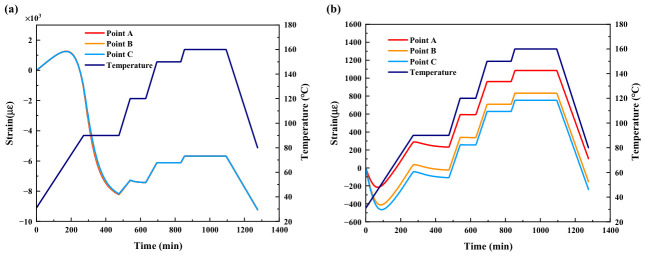
(**a**) Axial thermo-chemical strain during curing; (**b**) axial total strain during curing.

Figure 17a,b show the axial thermo-chemical strain and axial total strain at points A, B, and C. During the initial heating stage, the degree of cure is low, and thermo-chemical strain increases mainly due to thermal expansion. During the first holding stage, the resin undergoes rapid curing, and chemical shrinkage becomes dominant, which leads to a marked decrease in thermo-chemical strain. As curing approaches completion, the effect of shrinkage weakens and thermal strain becomes dominant, with strain varying in proportion to temperature. The thermo-chemical strain remains nearly identical at the inner and outer points due to the small temperature difference.

The total strain shows a decrease followed by an increase during the initial heating stage. At the early stage, the mold, which has high thermal conductivity, responds quickly to temperature changes. Thermal expansion of the outer mold compresses the composite, which produces compressive deformation in the central region. The outer regions experience greater compression than the inner region. Figure 17b shows that compressive strains at points B and C exceed that at point A. As temperature rises, thermal expansion of the composite increases and gradually overcomes the constraint imposed by the mold, which causes the strain to increase.

**Figure 18:**
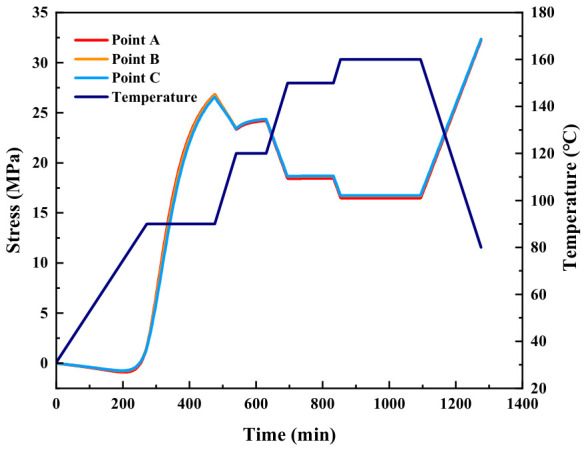
Axial stress changes during curing.

Figure 18 shows the axial stress evolution at the inner and outer points. The stress trends at these points are similar. Stress remains low during the initial stage of curing and increases sharply during the rapid curing stage. This increase results from a mismatch in curing shrinkage between inner and outer layers. Interlayer constraint restricts the axial shrinkage of the winding layers, which leads to the development of tensile stress. During subsequent heating stages, part of the axial stress is released. During cooling, axial stress increases again due to thermal contraction mismatch among layers caused by the temperature gradient. Resin shrinkage during curing and thermal contraction during cooling are the primary sources of residual stress.

### 4.2. Comparison of Simulation and Experiment

**Figure 19:**
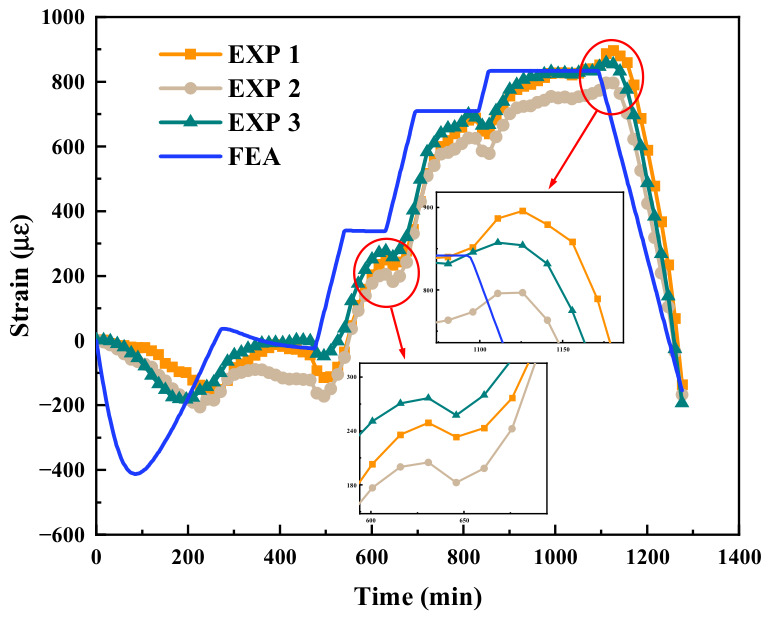
Comparison of experimental and simulated axial strain.

The axial strain data from the three ring specimens were averaged to obtain the experimental result. The FBG sensors were embedded at the location corresponding to point B in Figure 14, and the axial total strain at point B was taken as the simulation result. Figure 19 shows the comparison. The three experimental curves follow the same trend and are in close agreement, which indicates good repeatability.

During the initial heating stage, the compressive strain in the experimental results is smaller than that predicted by the simulation. The linear elastic constitutive model used in the simulation does not capture the viscoelastic behavior of the resin in the early curing stage. Resin flow reduces local compression. The composite is modeled as a homogeneous material, while the actual winding layer consists of multiple fiber tows with microscopic gaps and resin-rich regions. The central region is subjected to weaker mold constraint than that predicted by the model.

During the early cooling stage, the experimental strain increases relative to the simulation. The temperature sensor records surface temperature, while the internal temperature remains higher due to delayed heat transfer. The measured value of $\lambda^{T}$ in Equation (8) is lower than the actual value, which leads to an overestimation of axial strain. An opposite trend is observed during the transition from the holding stage to the heating stage. The experimental and simulation curves show consistent overall trends. At the end of curing, the residual axial strains from the experiments are −136.25 με, −167.08 με, and −193.75 με. The average value differs from the simulated result (−154.64 με) by 7.15%, which supports the validity of the numerical model.

## 5. Conclusions

Multi-field coupled numerical simulation and strain monitoring experiments were conducted for the curing process of resin-based carbon fiber composite rings. The main findings are summarized as follows.

1. Non-isothermal DSC testing of LD-2184 epoxy resin confirmed a single-stage autocatalytic curing reaction. Curing kinetic parameters were obtained through fitting. The elastic modulus of the cured resin was determined by uniaxial tensile testing. The coefficient of thermal expansion was obtained from CTE measurements.
2. A multi-field coupled curing model was established, which includes heat conduction, curing kinetics, and curing deformation. Numerical analysis shows the presence of a temperature gradient in the filament-wound layer due to heat conduction and the exothermic reaction. The temperature difference across the thickness remains small due to the thin structure. Thermo-chemical strain shows a similar distribution along the thickness direction. Total strain varies significantly under mold constraint. Resin chemical shrinkage and thermal contraction mismatch during cooling govern the development of residual stress.
3. Composite ring specimens with embedded FBG sensors were fabricated using a wet winding process. Strain was monitored during curing. The difference between measured and simulated residual axial strain is 7.15%, which supports the validity of the multi-field coupled model. The deviation is attributed to the absence of viscoelastic effects in the early curing stage, the homogenized representation of the winding layers, and the delayed thermal response within the composite.

## Figures and Tables

**Table 1:** Characteristic parameters of non-isothermal DSC test.

Heating Rate (°C·min^−1^)	Peak Temperature (°C)	Peak Heat Flow (mW·mg^−1^)	Total Heat Release (mJ·mg^−1^)
5	165.82	1.053	607.66
10	184.26	1.889	593.98
15	196.44	2.670	585.66
20	206.44	3.492	585.56

**Table 2:** Mechanical properties of T800 carbon fiber and LD-2184 resin.

Parameter	T800 Carbon Fiber	LD-2184 Resin
$E_{1}$/GPa	294	2.810
$E_{1}=E_{2}$/GPa	14	2.810
$G_{12}=G_{13}$/GPa	15	1.041
$G_{23}$/GPa	5.5	1.041
$\nu_{12}=\nu_{13}$	0.23	0.35
$\nu_{23}$	0.25	0.35
$\phi_{1}$/K^−1^	−9 × 10^−7^	6.060 × 10^−5^
$\phi_{2}=\phi_{3}$/K^−1^	7.2 × 10^−6^	6.060 × 10^−5^
V/%	50	50
$v_{sh}$	-	0.01695
k/(W·m^−1^·K^−1^)	0.742 + 9.02 × 10^−4^*T*	0.04184[3.85+(0.035T−0.141)α]
ρ/(kg·m^−3^)	1790	90α + 1232 (α≤0.45) 1272 (α≥0.45)
C/(J·kg^−1^·K^−1^)	1390 + 4.50*T*	$4184(0.468+5.975\times 10^{-4}T-0.141\alpha$)

**Table 3:** Mechanical properties of 30CrMnSiA steel.

Parameter	30CrMnSiA Steel
E/GPa	200
ν	0.33
k/(W·m^−1^·K^−1^)	29.3
ρ/(kg·m^−3^)	7800
C/(J·kg^−1^·K^−1^)	520
ϕ/K^−1^	1.172 × 10^−5^

**Table 4:** Mechanical properties of the composite at α=1.

$E_{1}$/GPa	$E_{1}=E_{2}$/GPa	$G_{12}=G_{13}$/GPa	$G_{23}$/GPa	$\phi_{1}$/K^−1^	$\phi_{2}=\phi_{3}$/K^−1^
148.417	6.082	2.326	2.644	−3.18 × 10^−7^	4.45 × 10^−5^

## Data Availability

The raw/processed data required to reproduce these findings cannot be shared at this time as the data also form part of an ongoing study.
